# Nitroxide-Mediated Controlled Radical Copolymerization of α-Trifluoromethylstyrenes with Styrenes

**DOI:** 10.3390/molecules29061214

**Published:** 2024-03-08

**Authors:** Tadashi Kanbara, Yuriko Ito, Airi Yamaguchi, Tomoko Yajima

**Affiliations:** Department of Chemistry, Ochanomizu University, 2-1-1 Otsuka, Bukyo-ku, Tokyo 104-8610, Japan; kanbara.tadashi@ocha.ac.jp (T.K.);

**Keywords:** fluorine-containing polymers, nitroxide-mediated polymerization, controlled polymerization

## Abstract

Fluorinated polymers are important materials in everyday life; however, most monomers of widely used fluoropolymers are gaseous, and their polymerization is difficult in an ordinary laboratory. Therefore, partially fluorinated polymers have recently been reported. As an easy-to-handle fluorine-containing monomer, α-trifluoromethylstyrene (TFMST) can be used to produce partially fluorinated polymers with trifluoromethyl groups in the main chain; however, TFMST does not homopolymerize, and there are limited reports on its copolymerization with styrene (ST). In this study, we applied the controlled radical polymerization method, which is effective for the polymerization of ST, to the copolymerization of TFMST and ST. We also showed that nitroxide-mediated polymerization is effective. The content ratio of TFMST in the TFMST–ST copolymer can be controlled between 10% and 40% by changing its monomer ratio. Additionally, the polymerization of TFMST and ST with substituents was performed to increase structural variations. The thermal stability as well as water and oil repellency of the synthesized polymers with different composition ratios and substituents were also evaluated.

## 1. Introduction

Fluorinated polymers are important materials in everyday life because of their unique properties, such as heat resistance, water repellency, and low dielectric constant [1,2,3,4]; however, well-established fluoropolymers, such as polytetrafluoroethylene, poly(vinylidenedifluoride), and poly(chlorotrifluoroethylene) are prepared from fluorinated olefin monomers that are gaseous, pose severe safety concerns [5,6], and are difficult to polymerize in an ordinary laboratory. Most of these polymers have very high melting points and poor solubility in common solvents and they lack formability [7,8]. Therefore, (semi)fluorinated polymers using fluorine-containing monomers, which are easy to handle, have recently attracted considerable attention [9]. Semifluorinated polymers include those with a fluoroalkylene in the main chain and those with a fluorine functional group in the polymer side chain. To incorporate fluoroalkylene into the backbone, the use of telechelic functional poly(perfluoroethers) [10] and leveraging difunctional perfluorinated monomers [9,11,12] have been reported. These polymerizations yield fluorine-substituted main chain polymers; however, the polymerization cannot be controlled [13]. In contrast, as examples of semifluorinated polymers with fluorine functional groups on the side chains, polymerization of fluorinated acrylate [14,15] or styrene (ST) [16,17] has been reported. Although it is easy to handle the monomer, the weakness of the main chain is a problem; therefore, strength—one of the advantages of fluoropolymers—cannot be realized. Here, when α-(trifluoromethyl)acrylate or α-trifluoromethylstyrene (TFMST) is used as a monomer, the tertiary hydrogen in the polymer main chain is replaced by a trifluoromethyl group. This displacement improves chemical resistance. Nevertheless, the polymerization of α-(trifluoromethyl)acrylate [18,19] and TFMST [20] is hindered by the presence of a bulky, electron-withdrawing trifluoromethyl group at the α-position. For instance, for TFMST, the kinetic study of radical copolymerization with ST was reported by Ito [20], and the monomer reactivity ratios of TFMST and ST revealed r_TFMST_ = 0.00 and r_ST_ = 0.60; therefore, TFMST did not homopolymerize. Although TFMST retards the polymerization rates, its copolymerization with ST proceeds. Ameduri reported controlled radical polymerization using iodine transfer polymerization (ITP) [21,22]. Despite the potential of the TFMST–ST copolymer, there was only one report on the controlled copolymerization of TFMST and ST; therefore, further synthetic studies were conducted. Three typical controlled radical polymerizations effective for ST, atom transfer radical polymerization (ATRP) [23,24,25], reversible addition-fragmentation chain transfer (RAFT) [26,27,28], and nitroxide-mediated polymerization (NMP) [29,30,31] were applied to the TFMST–ST copolymerization. Copolymerization of TFMST and ST substituted with trifluoromethyl groups has also been investigated. Although the copolymerization of TFMST–ST limits the number of trifluoromethyl groups introduced, the fluorine content of the polymer can be improved by introducing additional fluorine functional groups into the monomer. The thermophysical properties as well as water and oil repellency of the resulting polymers have also been evaluated.

## 2. Results and Discussion

First, copolymerization of ST and TFMST (90:10 mixture) was conducted using three different polymerization methods with high-ST-control capability (Table 1). All methods produced polymers with almost 9:1 incorporation of ST and TFMST. The composition ratio of ST to TFMST in the synthesized polymers was determined by comparing the integrated values of ^19^F NMR of CF_3_ of benzotrifluoride (BTF: internal standard) and TFMST (Figure S1 and Table S1).

When ATRP using (1-bromoethyl)benzene (**3b**) as an initiator and Cu-tetramethylethylenediamine (TMEDA) complex as a catalyst was investigated [32], the reaction proceeded to give a product with an M_n_ of 10,000, and a dispersity (*Đ* = M_w_/M_n_; M_w_ represents the weight-average molecular weight) of 1.30 was obtained (entry one). Then, RAFT polymerization was performed (entry two) [33]. The reaction was performed at 75 °C for 48 h using 2-cyano-2-propyl dodecyl trithiocarbonate (**3b**) as a chain transfer reagent and azobis(isobutyronitrile) (AIBN) as a radical initiator, yielding a product with an M_n_ of 2200. Because of the low molecular weight at 48 h, the reaction time was extended to 96 h but only an M_n_ of 4500 products was obtained in low yield (entry three). Then, we tried the NMP. The reaction was performed using Hawker’s universal initiator, *N*-*tert*-butyl-*N*-(2-methyl-1-phenylpropyl)-*O*-(1-pheylethyl)hydroxylamine (**3c**) [34], and polymerization proceeded but with low yield and M_n_ (entry four). We also performed the reaction using 2-methyl-2-[1-(diethoxyphosphinyl)-2,2-dimethylpropyl](*tert*-butyl)aminooxy]propanoic acid (BlocBuilder MA^®^ (**3d**)) [35,36] and found that the copolymer, which contains ST–TFMST = 91:9, was obtained in 56% yield with an M_n_ of 7300 and dispersity of 1.14 (entry five). Therefore, NMP with **3d** as the initiator was chosen for future studies.

NMP using different monomer ratios of ST and TFMST or α-methylstylene (MST) was investigated (Table 2). First, the polymerization of ST was performed to obtain polystyrene with an M_n_ of 8300 and a dispersity of 1.07 in 65% yield (entry 1). The polymerization of TFMST alone was also performed. However, the reaction did not proceed, confirming that it did not homopolymerize. Copolymerization with different ratios of ST and TFMST resulted in lower yields and increased dispersion as the ratio of TFMST increased (entries 2–5). The content of TFMST in the copolymer was at the same ratio when the ratio of ST–TFMST was 90/10. Nevertheless, as the content of TFMST increased, its content increased up to 40% but below the monomer content (Figure S2). Furthermore, the reaction rate and yield decreased as the ratio of TFMST increased. When equal amounts of ST and TFMST were used, an approximately 2:1 copolymer was produced. This result is consistent with the report on free radical polymerization by Ueda et al. [20]. These newly synthesized copolymers were soluble in organic solvents such as chloroform, THF, and toluene. As a control experiment, ST–MST copolymerization was also examined but MST was not introduced into the polymer chain at a 90:10 ratio (entry five). Copolymers with ST and MST contents of 7:3 were obtained when the monomer ratio was 50:50 (entry six).

The thermal stability of the resulting copolymers was then assessed by differential scanning calorimetry (DSC) and thermal gravimetric analysis. The 5% weight loss decomposition temperature (*T*_d5_) was found to be approximately 270 °C for all polymers. The glass transition temperature (*T*_g_) was lower for the ST–MST copolymer than for polystyrene but higher for polymers containing TFMST, with lower TFMST content. Although the polymer chain length synthesized here is relatively short, the copolymer of ST and TFMST tends to have higher *T*_g_ than polystyrene, while *T*_d5_ does not change significantly.

Thermogravimetric coupled with gas chromatography–mass spectrometry (TG-GC-MS) measurements were performed on copolymers with high TFMST content (40% TFMST content; Table 1, entry five) to investigate their degradation behavior (Figure S5). TG at a temperature between 30 and 600 °C was measured and the degradation products were observed by GC–MS. Based on the molecular weight, the degradation products derived from ST and TFMST were mainly observed, with less desorption of trifluoromethyl-derived degradation products. This result indicates that the depolymerization to ST and TFMST occurs without the elimination of the trifluoromethyl group. In addition, decomposition occurs in two stages, at around 280 and 410 °C. Since the ceiling temperature of polystyrene is 395 °C, the living end of the polymer is considered to be decomposed in the first peak.

**Figure 1:**
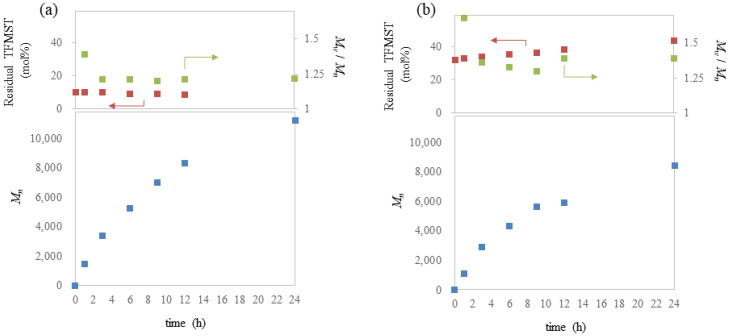
Plots of M_n_ (down; blue dot), residual amount of TFMST (up; red dot (mol%)), and M_w_/M_n_ (up; green dot) against reaction times; (**a**) ST to TFMST ratio of 90:10; (**b**) ST to TFMST ratio of 68:32.

Next, we followed the polymerization reaction over time (Figure 1, Tables S2 and S3). The M_n_ (blue dot), percentage of TFMST in the remaining monomer (mol%: red dot), and degree of *Đ* (green dot) were plotted against reaction times. When the monomer ratio of ST to TFMST was 90:10 (Figure 1a), the molecular weight increased over time, and the remaining percentage of TFMST remained constant. *Đ* was large in the early stages of the reaction but finally reached 1.2. When the monomer ratio is 68:32 (Figure 1b), the percentage of TFMST remaining in the monomer increases with time. This means that ST is preferentially consumed, and the monomer ratio changes during the reaction. As the ratio of TFMST increases during the reaction, the reaction rate decreases, and more TFMST is incorporated in the later stages of the reaction than in the earlier stages, which may result in a gradient of TFMST contents in the polymer chain.

The copolymerization of TFMST and the substituted ST were investigated (Table 3). Substituted ST and TFMST reacted in a ratio of 50:50 for 6 h, and a copolymer was obtained in all cases. The *p*-methoxystyrene-TFMST copolymer had a broad dispersity (entry one), whereas the *p*-trifluoromethylstyrene copolymer had a narrow dispersity, although the yield was low (entry two). When pentafluorostyrene was used, polymers with higher *T*_d5_ and *T*_g_ values than polystyrene were obtained (entry three).

Then, the copolymerization of substituted TFMST and ST was investigated (Table 4). TFMSTs with methyl, methoxy, and trifluoromethyl at the para position were synthesized according to protocols in the literature [37,38] (Scheme S1). As a result, copolymers of both monomers with electron-donating (entries one and two) and electron-withdrawing substituents (entry three) were obtained. Compared with the copolymers of unsubstituted TFMST and ST, *T*_d5_ did not change substantially but showed a higher *T*_g_ in all cases, especially for the copolymer with the pentafluoro group **5da**.

**Figure 2:**
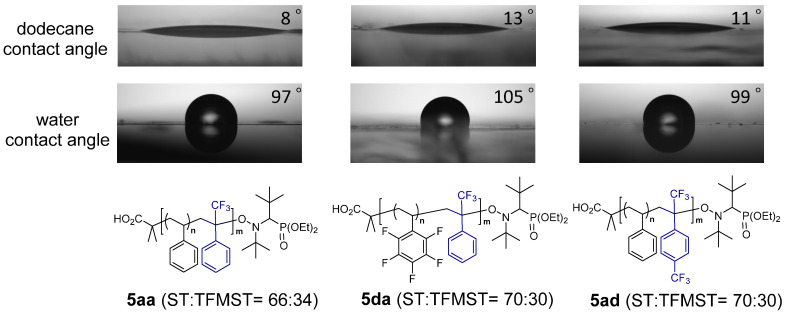
Water and oil contact angles of copolymers **5aa**, **5da**, and **5ad**.

Finally, we measured the water and oil contact angles of the synthesized copolymers (Figure 2, Tables S4–S6). A copolymer ST and TFMST (66:34) had a water contact angle of 97°. This value is smaller than that reported by Ameduri [13]; however, the difference may be because their polymer was synthesized by ITP and has a perfluorohexyl group at the polymer end. The oil repellency measured using dodecane as oil was 8°. Copolymers with different polymer ends (Table S4) and different ratios of ST to TFMST (Table S5) had no substantial effect on water and oil repellency, even with increasing fluorine content. The substituted copolymers were also measured (Table S6, Figure 2). The results for **5aa**, **5da** with a fluorine functional group on the ST monomer, and **5ad** with a trifluoromethyl group on the TFMST monomer were also shown (Figure 2). As a result, a slight improvement in water and oil repellency was observed when pentafluorophenyl groups were present [39].

## 3. Materials and Methods

### 3.1. General Information

#### 3.1.1. Materials

ST (99%) was purchased from Yoneyama Yakuhin Kogyo Co., Ltd. (Osaka, Japan). The chemicals 4-methoxystyrene (>98%), 4-(trifluoromethyl)styrene (>98%), 2-bromo-3,3,3-trifluoropropene (>97%), phenylbronic acid (>97%), 4-methylphenylbronic acid (>97%), 4-methoxyphenylbronic acid (>97%), 4-(trifluoromethyl)phenylbronic acid (>97%), and benzotrifluoride (>98%) were purchased from TCI (Mountain View, CA, USA). Chemicals such as 2,3,4,5,6-pentafluorostyrene (+97%) and AIBN were purchased from Wako (Richmond, VA, USA), while α-methylstyrene (98%) was purchased from Nacalai tesque (Kyoto, Iapan). *N*-tert-butyl-*N*-(2-methyl-1-phenylpropyl)-*O*-(1-phenylethyl)hydroxylamine (>98%) was purchased from Santa Cruz Biotechnology, Inc. (Dallas, TX, USA), and stored in the dark at −28 °C. BlocBuilder MA was supplied from Arkema (Colombes, France) and kept in the dark at 0 °C. Potassium carbonate (K_2_CO_3_) was purchased from Takahashi Pure Chemical Co. (Yokohama, Japan). Bis(triphenylphosphine)palladium(II) dichloride (PdCl_2_(PPh_3_)_2_) (97%) and ammonium chloride (NH_4_Cl) (99.5%) were purchased from Kanto Chemical Co., Inc. (Tokyo, Japan). The styrene monomers of 4-methoxystyrene, 4-(trifluoromethyl)styrene, and 2,3,4,5,6-pentafluorostyrene were used after removal of the polymerization inhibitor.

#### 3.1.2. General Measurements

^1^H NMR and ^19^F NMR were measured on a JEOL GSX-400 spectrometer (JEOL, Akishima, Japan, 400 MHz for ^1^H, 101 MHz for ^13^C, and 376 MHz for ^19^F) and a JEOL ECX-500 spectrometer (JEOL, Akishima, Japan, 500 MHz for ^1^H, 126 MHz for ^13^C, and 471 MHz for ^19^F). All chemical shifts are reported as n parts-per-million downfield of the standards. Tetramethylsilane ^1^H 0.0 was used as the internal standard for chloroform-*d* solutions. The values of the number-average molecular weight (M_n_) and polydispersity (M_w_/M_n_) were estimated by gel permeation chromatography in THF calibrated with polystyrene as standard, operating at 1.0 mL/min with an L-2490 refractive index detector, two Showdex KF-803L columns (8.0 × 300 mm), and KF-806L (8.0 × 300 mm) at 40 °C. THF was purchased from Kanto Chemical Co., Inc.

#### 3.1.3. Thermal Measurements

Thermogravimetric analysis of the polymers was performed on a BRUKER TG-DTA2000SA (Bruker, Billerica, MA, USA) under nitrogen conditions at 10 °C min^−1^ from 0 °C to 500 °C. DSC was measured on a BRUKER DSC100SA under nitrogen conditions at 10 °C min^−1^ from −30 °C to 250 °C. The procedure of thermogravimetric (TG) (NETZSCH STA2500 Regulus, Netzsch, Selb, Germany) coupled with gas chromatography–mass spectrometry (GC/MS) was used to carry out pyrolysis/combustion experiments. In total, a 1.3 mg sample was pyrolyzed/combusted in the TG at temperatures between 30 °C and 600 °C with a heating rate of 10 °C/min. N_2_ gas with a flow rate of 100 mL/min was used as the carrier gas to create an inert atmosphere in the pyrolysis process. The evolved gases at a target temperature (300 °C for an inert atmosphere that corresponds to the maximum mass loss rate) were sampled via an autoinjector system that was connected to the TG system. The remainder of the gas was purged to the atmosphere. All transfer lines of the autoinjector were maintained at 300 °C to avoid any condensation of volatile gases whose boiling temperature was less than 300 °C. Gas analysis was carried out with gas chromatography–mass spectrometry (GC/MS) (Agilent 8890 GC system JMS-Q1600GC (Agilent, Santa Clara, CA, USA) for GC, JMS-Q1500GC Master-Quad GC/MS (JEOL, Akishima, Japan) for MS). The GC instrument was equipped with HP-5MS (5%-phenyl)-methylpolysiloxane nonpolar (15 m length, 0.25 mm I.D., and 0.25 μm film) and an HP-MOLESIEVE (30 m length, 0.53 mm I.D) column. The initial oven temperature of 40 °C was kept isothermal for 3 min, then heated to 600 °C at a rate of 10 °C/min, and held at 600 °C for 5 min. The temperatures of the MS source and MS quad were 300 and 150 °C, respectively. The detector consists of a mass selective detector, and electron impact mass spectra were acquired with 70 eV ionizing energy with a scanning range from 10 to 1000 Da and with a scan rate of 1 scan s^−1^. The MS transfer line and the ion source temperature were maintained at 300 °C.

#### 3.1.4. Water and Oil Repellency Measurements

The polymer film obtained for the contact angle assessments was formed using a spin coater (Mikasa Opticoat MS-A100 model, Tokyo, Japan). A solution of 100 mg of the gained polymer in 270 µL of toluene (30 wt%) was placed on a 70 × 40 mm slide glass. The substrate surface was covered with the dissolved polymer using a pipette, followed by spinning at 100–400 rpm for 20 s to spread and form a uniform thin film. The film sample was then heated at 110 °C for 6 min and left overnight. The contact angle of the film sample was measured using a Nick LSE-ME5 (Nick, Saitama, Japan) contact angle measurement device. Measurements were performed at least three different sites, and the final value was averaged. A drop of water (5 μL) or dodecane (6 μL) was deposited using a microsyringe. The angles were determined using the Laplace–Young equation approximately 1 s after the drop deposition.

### 3.2. General Procedure for Synthesis of Substituted TFMST

To a 100 mL flask equipped with a magnetic stir bar, arylbronic acid (8 mmol), 2-bromo-3,3,3-trifluoropropene (1.65 mL, 16 mmol), dry THF (24 mL), K_2_CO_3_ (2.0 M, 16 mL), and PdCl_2_(PPh_3_)_2_ (168 mg, 0.24 mmol) was added. The mixture was frozen–degassed with liquid nitrogen and vacuum evacuation (×3). After dissolution, the reaction vessel was injected with argon. The resulting solution was stirred at 65 °C for 24 h. After the reaction mixture was cooled to room temperature, the mixture was quenched with saturated aqueous NH_4_Cl, extracted with Et_2_O, dried over Na_2_SO_4_, filtered, and concentrated under pressure. After removing the solvent, the crude product was purified by distillation under reduced pressure (pressure: 20 mbar, bath temperature: 90 °C) to obtain a colorless oil. Compounds were identified according to the literature [36,37].

### 3.3. General Procedure for Copolymerization

#### 3.3.1. ATRP Polymerization

To a 10 mL flask equipped with a magnetic stir bar, **3a** (18.5 mg, 0.1 mmol), Cu-TMEDA (46.4 mg, 0.1 mmol), and monomers (styrenes and TFMSTs, 10 mmol) were added. The mixture was frozen–degassed with liquid nitrogen and vacuum evacuation (×3). After dissolution, the reaction vessel was injected with Ar. The resulting solution was stirred at 110 °C for 12 h. After cooling the reaction mixture to room temperature, the mixture was dissolved in a small amount of CH_2_Cl_2_ and precipitated in cold hexane. The resulting polymer was dried under a vacuum.

#### 3.3.2. RAFT Polymerization

To a 10 mL flask equipped with a magnetic stir bar, **3b** (34.6 mg, 0.1 mmol), AIBN (4.1 mg, 0.025 mmol) in 1,4-dioxane (2.5 mL), and monomers (styrenes and TFMSTs, 10 mmol) were added. The mixture was frozen–degassed with liquid nitrogen and vacuum evacuation (×3). After dissolution, the reaction vessel was injected with Ar. The resulting solution was stirred at 75 °C. After cooling the reaction mixture to room temperature, the mixture was dissolved in a small amount of CH_2_Cl_2_ and precipitated in cold hexane. The resulting polymer was dried under a vacuum.

#### 3.3.3. NMP Polymerization

BlocBuilder MA (**3d**) (38.1 mg, 0.1 mmol) and monomers (ST and TFMSTs, 10 mmol) were added to a 10 mL flask equipped with a magnetic stir bar. The mixture was frozen–degassed with liquid nitrogen and vacuum evacuation (×3). After dissolution, the reaction vessel was injected with Ar. The resulting solution was stirred at 110 °C for 6 h. After cooling the reaction mixture to room temperature, the mixture was dissolved in a small amount of CH_2_Cl_2_ and precipitated in cold hexane. The resulting polymer was dried under a vacuum.

## 4. Conclusions

In this study, controlled copolymerization of TFMST and ST was investigated. We found that NMP using BlocBuilder MA (**3d**) as the initiator yielded the TFMST–ST copolymer in low dispersity (*Đ)*. The content ratio of TFMST can be controlled between 10% and 40% by changing its monomer ratio. Additionally, TFMST and ST with their substituents were polymerized to obtain novel copolymers. Thermal measurements of the synthesized copolymers were performed. The resulting copolymers exhibited lower *T*_d5_ than polystyrene, except for those containing 10% TFMST. All copolymers exhibited a higher *T*_g_ than polystyrene, especially for the copolymer with the pentafluoro group **5da**. Moreover, a slight improvement in the water and oil contact angle was observed with the copolymer with pentafluoro groups of **5da**.

## Figures and Tables

**Table 1:** Reactions using different polymerization methods.

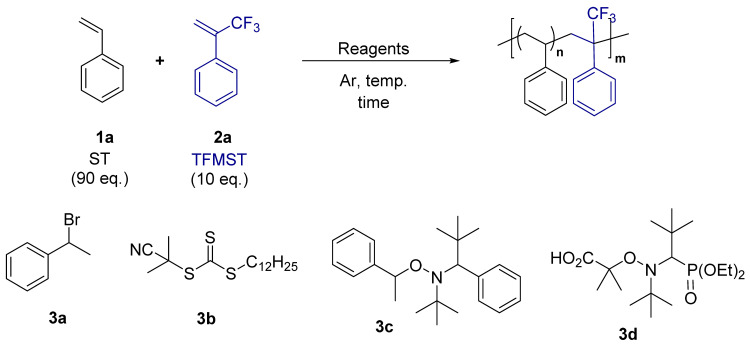								
**Entry**	**Reagents**	**Temp.** **[°C]**	**Time** **[h]**	**Yield ^(a)^** **[%]**	**Ratio of ST ^(b)^** **[%]**	**Ratio of TFMST ^(b)^** **[%]**	**M_n_ ^(c)^**	**M_w_/M_n_ ^(c)^**
1 ^(e)^	**3a** (1 eq.), Cu-TMEDA (1 eq.)	110	12	62	91	9	10,000	1.30
2 ^(f)^	**3b** (1 eq.), AIBN (0.25 eq.) 1,4-dioxane	75	48	28 ^(d)^	90	10	2200	1.27
3 ^(f)^	**3b** (1 eq.), AIBN (0.25 eq.) 1,4-dioxane	75	96	21	87	13	4500	1.10
4 ^(g)^	**3c** (1 eq.)	110	6	16	90	10	2800	1.18
5 ^(g)^	**3d** (1 eq.)	110	6	56	91	9	7300	1.14

^(a)^ Cold hexane-insoluble part; ^(b)^ determined by ^19^F NMR using BTF as an internal standard after purification; ^(c)^ determined by gel permeation chromatography in THF based on linear polystylene as calibration standard; ^(d)^ concentrated residue; ^(e)^ the mixture of **1a** (9 mmol), **2a** (1 mmol), **3a** (0.1 mmol), and Cu-TMEDA (0.1 mmol) under an Ar atmosphere was stirred at 110 °C for 12 h; ^(f)^ the mixture of **1a** (9 mmol), **2a** (1 mmol), **3b** (0.1 mmol), and AIBN (0.025 mmol) in 1,4-dioxane (2.5 mL) under an Ar atmosphere was stirred at 75 °C for 48 or 96 h; ^(g)^ the mixture of **1a** (9 mmol), **2a** (1 mmol), and **3c** or **3d** (0.1 mmol) under an Ar atmosphere was stirred at 110 °C for 6 h.

**Table 2:** Reaction using different ratios of ST vs. TFMST or MST ^(a)^.

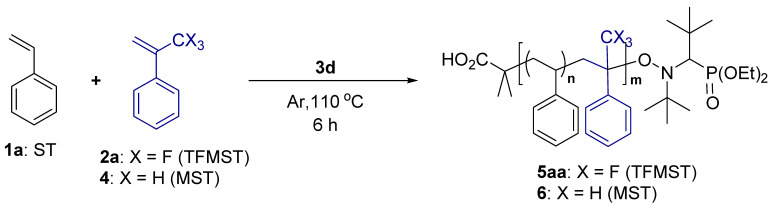									
**Entry**	**ST (1a)** **[eq.]**	**2a or 4** **[eq.]**	**Yield ^(a)^** **[%]**	**Ratio of ST** **[%]**	**Ratio of (TF)MST** **[%]**	**M_n_ ^(b)^**	**M_w_/M_n_ ^(b)^**	***T*** **_d5%_** **[** **°** **C]**	***T*** **_g_** **[** **°** **C]**
1	100	-	65	100	-	8300	1.07	275	71
2	90	**2a** (10)	56	91	9 ^(c)^	7300	1.14	285	103
3	70	**2a** (30)	54	82	18 ^(c)^	8300	1.31	279	107
4	50	**2a** (50)	32	66	34 ^(c)^	7400	1.30	261	80
5	30	**2a** (70)	20	60	40 ^(c)^	6100	1.47	261	91
6 ^(d)^	90	**4** (10)	47	100	0	7100	1.11	-	-
7 ^(e)^	50	**4** (50)	27	71	29 **^(^**^f)^	7500	1.34	278	65

^(a)^ Cold hexane-insoluble part; ^(b)^ determined by gel permeation chromatography in THF based on linear polystyrene as calibration standard; ^(c)^ determined by ^19^F NMR using BTF as an internal standard after purification; ^(d)^ 8 h; ^(e)^ 22 h; ^(f)^ determined by ^1^H NMR.

**Table 3:** Reaction using substituted ST ^(a)^.

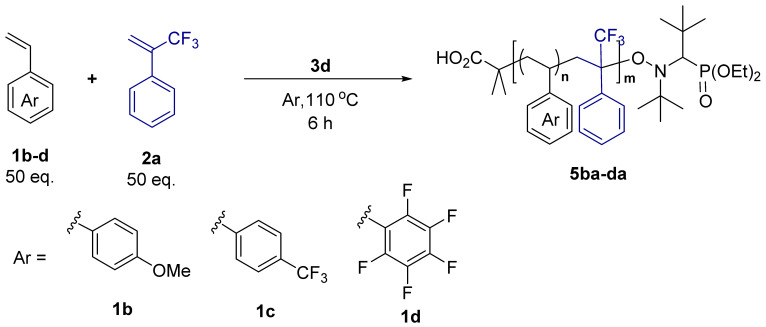									
**Entry**	**ST**	**Yield ^(b)^** **[%]**	**Copolymer**	**Ratio of ST ^(c)^** **[%]**	**Ratio of TFMST ^(c)^** **[%]**	**M_n_ ^(d)^**	**M_w_/M_n_**	***T*** **_d5%_** **[** **°** **C]**	***T*** **_g_** **[** **°** **C]**
1	**1b**	26	**5ba**	69	31	5800	1.50	252	100
2	**1c**	19	**5ca**	77	23	4700	1.18	238	94
3	**1d**	35	**5da**	70	30	9300	1.49	293	130

^(a)^ The mixture of **1b**, **c**, or **d** (5 mmol), **2a** (5 mmol), and **3c** (0.1 mmol) under an Ar atmosphere was stirred at 110 °C for 6 h; ^(b)^ cold hexane-insoluble part; ^(c)^ determined by ^19^F NMR using BTF as an internal standard after purification; ^(d)^ determined by gel permeation chromatography in THF based on linear polystyrene as calibration standard.

**Table 4:** Reaction using substituted MST ^(a)^.

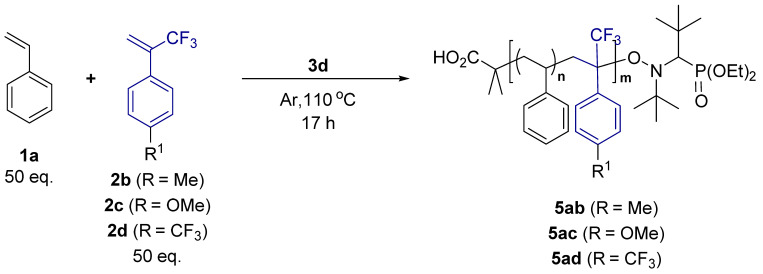									
**Entry**	**TFMST**	**Yield ^(b)^** **[%]**	**Copolymer**	**Ratio of ST ^(c)^** **[%]**	**Ratio of TFMST ^(c)^** **[%]**	**M_n_ ^(d)^**	**M_w_/M_n_ ^(d)^**	***T*** **_d5%_** **[** **°** **C]**	***T*** **_g_** **[** **°** **C]**
1	**2b**	20	**5ab**	73	27	8600	1.24	251	107
2	**2c**	33	**5ac**	68	32	6100	1.47	255	93
3	**2d**	46	**5ad**	70	30	10,000	1.37	278	110

^(a)^ The mixture of **1a** (5 mmol), **2b**, **c**, or **d** (5 mmol), and **3c** (0.1 mmol) under an Ar atmosphere was stirred at 110 °C for 17 h; ^(b)^ cold hexane-insoluble part; ^(c)^ determined by ^19^F NMR using BTF as an internal standard after purification; ^(d)^ determined by gel permeation chromatography in THF based on linear PSt as calibration standard.

## Data Availability

The data presented in this study are available in the Supplementary Materials.

## Supplementary Materials

The following supporting information can be downloaded at https://www.mdpi.com/article/10.3390/molecules29061214/s1, Scheme S1: synthesis of monomers; Figure S1: example of ^19^F NMR spectra (CDCl_3_) of synthesized polymer; Table S1: the data used to determine the molar composition of the polymers; Figure S2: TFMST ratio in monomer vs. polymer; Table S2: time course of polymerization using 90 mol% of ST; table S3: time course of polymerization using 68 mol% of ST; Figure S3: TG curves with different TFMST content; Figure S4: TG curves with different substituent; Figure S5: TG-GC-MS in mass spectrum; Table S4: static contact angle measurements of polymers with different polymerization methods; Table S5: static contact angle measurements of polymers with different ratios of TFMST; Table S6: Static contact angle measurements of polymers with different Ar^1^ or Ar^2^. Refs. [40,41,42] are cited in the Supplementary Materials.
