# Supplementary material for: Nitroxide-Mediated Controlled Radical Copolymerization of α-Trifluoromethylstyrenes with Styrenes
Source: Molecules. 2024 Mar 8;29(6):1214. doi: 10.3390/molecules29061214 (PMC10975655; doi:10.3390/molecules29061214)
Supplement: Supplementary file 1 [file molecules-29-01214-s001.zip › molecules-2885605-supplementary.pdf]

Supporting Information

Nitroxide-Mediated Controlled Radical Copolymerization of α-Trifluoromethylstyrenes with styrenes

Tadashi Kanbara, Yuriko Ito, Airi Yamaguchi, Tomoko Yajima

Department of Chemistry, Ochanomizu University, 2-1-1, Otsuka, Bunkyo-ku, Tokyo 112-8610, Japan

Table of contents

1. Synthesis of monomers S2
2. Determination of ST and TMSMST composition ratio in copolymers S3
3. Plot of TFMST ratio of monomer vs polymer. S5
4. Time course of copolymerization S6
5. TG curves S7
6. TG-GC-MS S8
7. Water and oil repellency S9

1. **Synthesis of monomers**

Monomers were synthesized according to the literature. [1,2]

To a 100 mL flask equipped with a magnetic stir bar was added arylbronic acid (8 mmol), 2-bromo-3,3,3-trifluoropropene (1.65 mL, 16 mmol), dry THF (24 mL), K_2CO_3 (2.0 M, 16 mL) and $PdCl_2(PPh_3)_2$ (168 mg, 0.24 mmol). The mixture was frozen-degassed with liquid nitrogen and vacuum evacuation (× 3). After dissolution, the reaction vessel was injected argon. The resulting solution was stirred at 65 °C for 24 h. After reaction mixture was cooled to room temperature, the mixture was quenched with saturated aqueous NH_4Cl, extracted with Et_2O, dried over Na_2SO_4, filtered, and concentrated under pressure. After removing the solvent, the crude product was purified by distillation under reduced pressure (pressure: 20 mbar, bath temperature: 90 °C) to obtain a colorless oil. Compounds were identified according to the literature. [3-5]

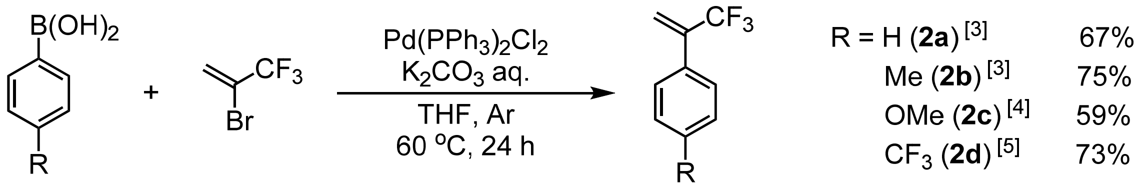

Scheme S1. Synthesis of monomers.

[1] Liu Y., Zhou Y., Zhao Y., Qu J., *Org. Lett.*, **2017**, *19*, 946−949.

[2] J. Walkowiak-Kulikowska, F. Boschet, G. Kostov, V. Gouverneur, B. Ameduri, *Eur. Polym. J.*, **2016**, *84*, 612–621.

[3] R. Pan, X. Liu, M. Deng, *J, Fluor. Chem.*, **1999**, *95*, 167−170.

[4] Miura T., Ito Y., Murakami M., *Chem. Lett.*, **2008**, *37*, 1006−1007.

[5] Nader B. S., Cordova J. A., Reese K. E., Powell C. L., *J. Org. Chem.,* **1994**, *59*, 2898−2901.

2. Determination of ST and TFMST composition ratio in copolymers

Using a known weight of benzotrifluoride (BTF) as an internal standard for ^{19}F NMR studies, ST and TFMST composition ratio was calculated by comparison of the integrated values of CF_3 of BTF and CF_3 of TFMST in copolymer.

Mol% of TFMST in the copolymer $\%_{TFMST} = A * 100$

Mol% of ST in the copolymer $\%_{ST} = (1\text{-}A) * 100$

With

$$\frac{m_{copolym} * \dfrac{M_{TFMST} \times A}{M_{TFMST} \times A + M_{ST} \times (1 - A)}}{M_{TFMST}} = n_{TFMST}$$

Where

$$n_{TFMST} = n_{BTF} \times \frac{I_{copolym}}{I_{BTF}}$$

$m_{copolym}$ Measured polymer weight (mg)

M_{TFMST}, M_{ST} : Molecular weigh

n_{TFMST}, n_{BTF} (mmol)

$I_{copolym}$, I_{BTF}: Integrated value

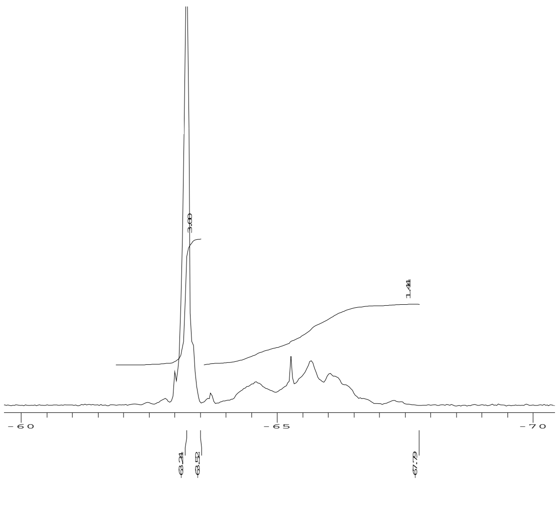

Figure S1. Example of ^{19}F NMR spectra ($CDCl_3$) of synthesized polymer.

Polymer	$m_{copolym}$ [mg]	M_{TFMST}	M_{ST}	n_{TFMST} [mmol]	n_{BTF} [mmol]	$I_{copolym}$	I_{BTF}	A
Table 2, Entry 2	137.2	172.15	104.15	0.120	0.140	2.50	3.00	0.090
Table 2, Entry 3	13.6	172.15	104.15	0.0206	0.0712	0.87	3.00	0.176
Table 2, Entry 4	11.7	172.15	104.15	0.0309	0.0643	1.44	3.00	0.335
Table 2, Entry 5	14.1	172.15	104.15	0.0430	0.121	1.07	3.00	0.400
Table 3, Entry 1	21.6	172.15	134.18	0.0462	0.0671	2.07	3.00	0.310
Table 3, Entry 2	11.0	172.15	172.15	0.0147	0.107	3.00	21.78	0.230
Table 3, Entry 3	35.5	172.15	194.10	0.0562	0.0958	1.76	3.00	0.297
Table 4, Entry 1	10.2	186.17	104.15	0.0221	0.120	0.55	3.00	0.270
Table 4, Entry 2	14.9	202.17	104.15	0.0348	0.0704	1.48	3.00	0.315
Table 4, Entry 3	12.8	240.15	104.15	0.0266	0.107	3.00	12.14	0.301

Table S1. The data used to determine the molar composition of the polymers.

3. Plot of TFMST ratio of monomer vs polymer.

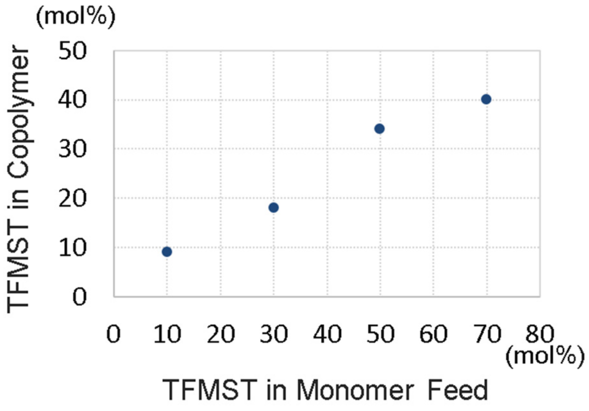

Figure S2. TFMST ratio in monomer vs polymer.

4. Time course of copolymerization.

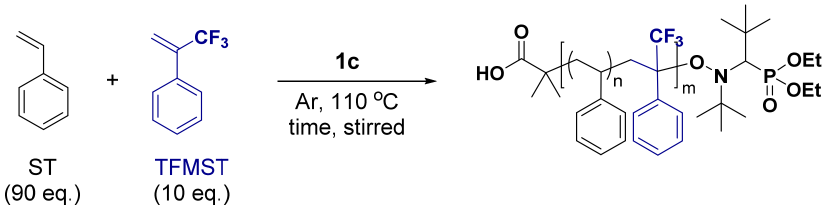

time	M_n a) (x 10^3)	M_w / M_n a)	Residual Monomer Ratio (mol%) b)	
			ST	TFMST
0 h	-	-	90	10
1 h	1.5	1.39	90	10
3 h	3.4	1.21	90	10
6 h	5.2	1.21	91	9
9 h	7.0	1.20	91	9
12 h	8.3	1.21	91	9
24 h	11.2	1.22	n.d.	n.d.

a) Determined by GPC in THF, based on linear PSt as calibration standards. b) Determined by ^{1}H NMR.

Table S2. Time course of polymerization using 90 mol% of ST.

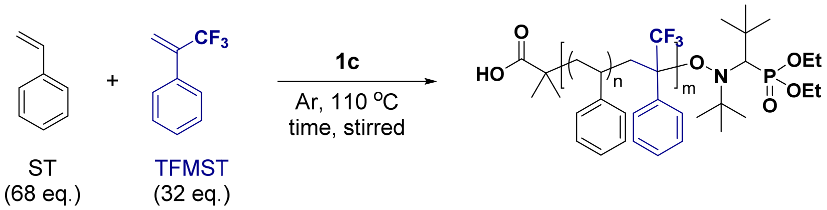

time	M_n a) (x 10^3)	M_w / M_n a)	Residual Monomer Ratio (mol%) b)	
			ST	TFMST
0 h	-	-	68	32
1 h	1.1	1.68	67	33
3 h	2.9	1.36	66	34
6 h	4.3	1.33	65	35
9 h	5.6	1.30	64	36
12 h	5.9	1.39	62	38
24 h	8.4	1.39	56	44

a) Determined by GPC in THF, based on linear PSt as calibration standards. b) Determined by ^{1}H NMR.

Table S3. Time course of polymerization using 68 mol% of ST.

5. TG curves.

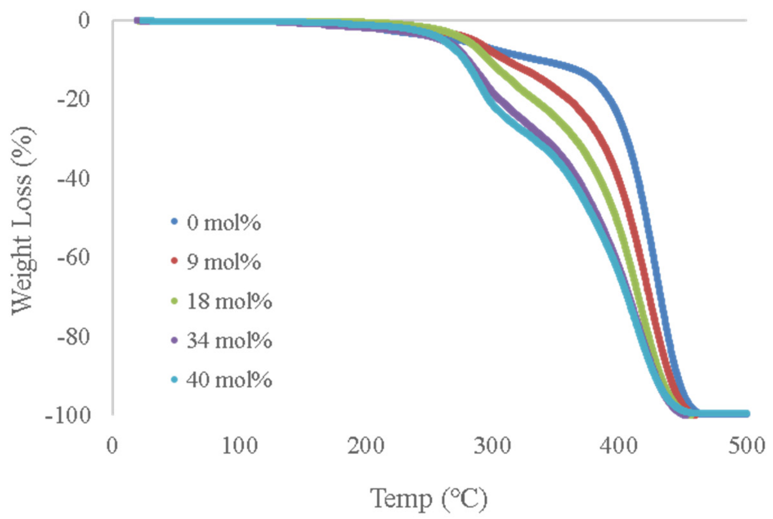

Figure S3. TG curves with different TFMST content.

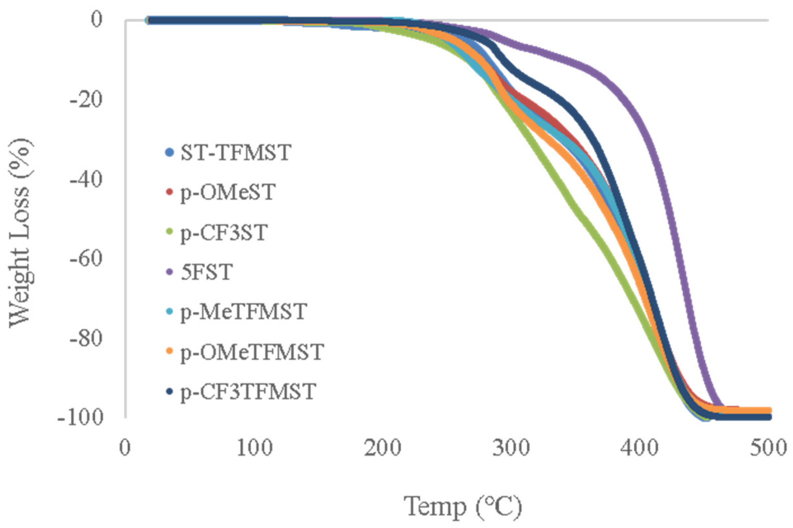

Figure S4. TG curves with different substituent.

6. TG-GC-MS

Procedure of thermogravimetric (TG) (NETZSCH STA2500 Regulus) coupled with a gas chromatography-mass spectrometry (GC/MS) was used to carry out pyrolysis/combustion experiments. 1.3 mg sample (Table 2, entry 5) is pyrolyzed/combusted in the TG at temperatures between 30 °C and 600 °C with a heating rate of 10 °C/min. N_2 gas with a flow rate of 100 mL/min was used as the carrier gas to create an inert atmosphere in pyrolysis process. The evolved gases at a target temperature (300 °C for inert atmosphere that corresponding to the maximum mass loss rate) was sampled via an autoinjector system that is connected with the TG system. The remainder of the gas is purged to the atmosphere. All transfer lines of the autoinjector are maintained at 300 °C to avoid any condensation of volatile gases whose boiling temperature is less than 300 °C. Gas analysis is carried out with a gas chromatography-mass spectrometry (GC/MS) (Agilent 8890 GC system JMS-Q1600GC for GC, JMS-Q1500GC Master-Quad GC/MS for MS). The GC instrument is equipped with HP-5MS (5%-phenyl)-methylpolysiloxane nonpolar (15 m length, 0.25 mm I.D. and 0.25 µm film) and HP-MOLESIEVE (30 m length, 0.53 mm I.D) column. The initial oven temperature of 40 °C is kept isothermal for 3 min, then heated to 600 °C at a rate of 10 °C/min, and held at 600 °C for 5 min. The temperatures of the MS source and MS quad are 300 and 150 °C, respectively. The detector consists of a mass selective detector and electron impact mass spectra are acquired with 70 eV ionizing energy with a scanning range from 10 to 1000 Da and with a scan rate of 1 scans s^{-1}. The MS transfer line and the ion source temperature are maintained at 300 °C. The chromatographic peaks are identified according to the National Institute of Standards and Technology (NIST) mass spectral data library.

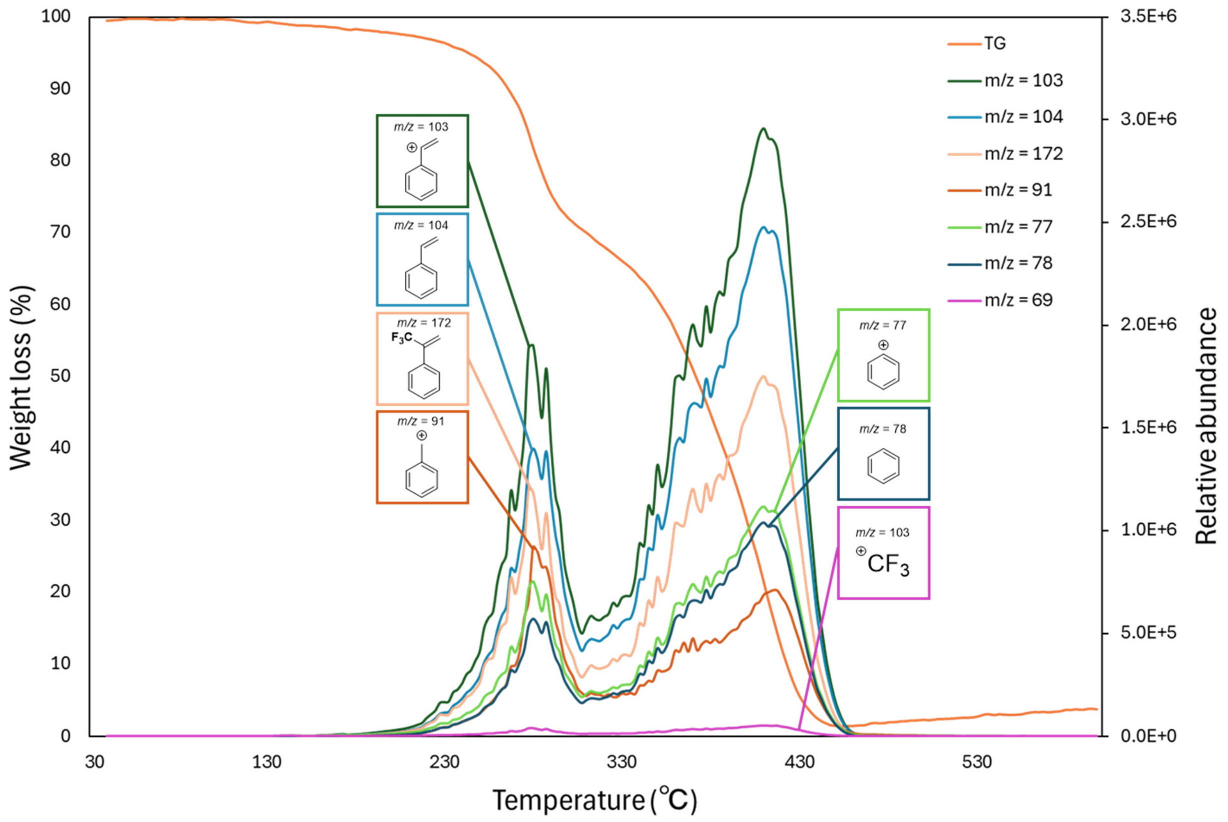

Figure S5. TG-GC-MS in mass spectrum.

7. Water and oil repellency.

Fluorine content was calculated using the following formula.

$$Fluorine\ content\ (\%) = \frac{(M_{\mathrm{n}} - M_{terminal\ site}) \times \frac{N_{fluorine} \times M_{fluorine}}{M_{unit}}}{M_{\mathrm{n}}}$$

Where

$M_{terminal\ site}$:Molecular weight of terminal site of polymers

$N_{fluorine}$:Numer of fluorine atoms per unit

$M_{fluorine}$:Molecular weight of fluorine

M_{unit} :Molecular weight of unit

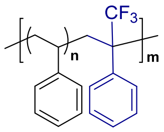

Polymerization methods	Composition of polymer		Fluorine content (wt%)	Contact angle (degree)	
	ST (%)	TFMST (%)		Water	Dodecane
ATRP	91	9	5	88	8
RAFT	87	13	6	93	10

Table S4. Static contact angle measurements of polymers with different polymerization methods.

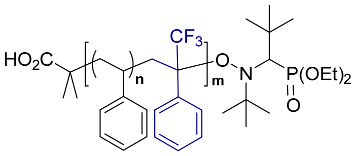

Composition of polymer		Fluorine content (wt%)	Contact angle (degree)	
ST (%)	TFMST (%)		Water	Dodecane
100	0	0	88	6
91	9	5	92	6
82	18	9	92	8
66	34	15	97	8
60	40	17	93	8

Table S5. Static contact angle measurements of polymers with different ratio of TFMST.

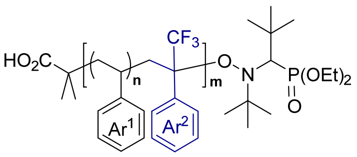

Ar^1	Ar^2	Composition of polymer Ar^1:Ar^2	Fluorine content (wt%)	Contact angle (degree)	
				Water	Dodecane
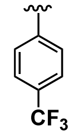	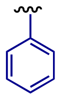	77 : 23	32	97	10
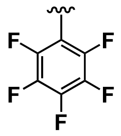	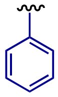	70 : 30	44	105	13
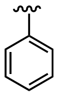	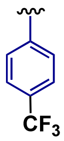	70 : 30	23	99	11

Table S6. Static contact angle measurements of polymers with different Ar^1 or Ar^2.
